# Heavy metals in vegetables: screening health risks involved in cultivation along wastewater drain and irrigating with wastewater

**DOI:** 10.1186/s40064-016-2129-1

**Published:** 2016-04-19

**Authors:** Ashita Sharma, Jatinder Kaur Katnoria, Avinash Kaur Nagpal

**Affiliations:** Department of Botanical and Environmental Sciences, Guru Nanak Dev University, Amritsar, Punjab 143005 India

**Keywords:** Heavy metals, Hazard quotient, Metal pollution index, Vegetables, Wastewater

## Abstract

Irrigation of agricultural land with wastewater leads to continuous buildup of metals at these sites which gets accumulated in the vegetables and crops growing on these sites. Not just the crops irrigated with wastewater are hazardous, in present study, we have found that vegetables growing in vicinity of wastewater drain are also not safe for human consumption. The risk associated with consumption of vegetables was assessed by calculating hazard quotient and results revealed that the hazard quotient for leafy and tuberous vegetables was higher than the safe limits in all the sites irrespective of mode of irrigation. Spinach was the most hazardous among all as the hazard quotient with respect to cobalt and copper was highest in spinach. Uptake trend of metals in all vegetables: *Iron* > *Cobalt* > *Copper* > *Cadmium* > *Lead.* Cadmium, a potential carcinogen was found in concentrations higher than permissible limits in many vegetables from all sites. Highest level of cadmium (1.20 mg/kg) and copper (81.33 mg/kg) was reported in site which was in vicinity of waste water drain but irrigated with ground water. Concentration of copper and lead in vegetable samples from different sites exhibited no statistically significant difference with respect to different sites.

## Background

Injudicious use of resources and haphazard urbanization have over-exploited the natural resources and caused detrimental effects on the environment. Unplanned economic development has led to pressure on cultivable land and suitable water for irrigation. To meet the food demands of exponentially growing human population, cultivation of food crops is carried out at places which are not suitable for agriculture like along wastewater drains or other polluted sites. To address water crisis, irrigation using the large amount of wastewater discharged from the rapid growing industries is being carried out in many parts of the world. Wastewater used for irrigation has many contaminants mainly heavy metals depending upon the source of discharge (Huibers and Van Lier 2005; Pedrero et al. 2010). In most of the developing countries, the wastewater discarded from industrial or residential areas is not treated and even, if it is treated the treatment process involves only primary processes which generally, do not remove heavy metals from the water. Long term use of wastewater for irrigation can cause accumulation of these metals in soil which can be further translocated to food crops and thus enter food chain (Arora et al. 2008; Gupta et al. 2010; Singh and Agrawal 2010). Though, such cultivation strategies help in addressing the issues of unemployment and increase the crop yield but the global food security is not attained. According to World Health Organization (WHO), food security is achieved when “everyone and always” have access to “sufficient and safe” food. Intake of metals through diet is reported across the globe and health hazards associated with these metals are also known (Arora et al. 2008; Orisakwe et al. 2012; Petroczi and Naughton 2009; Singh et al. 2010; Zhuang et al. 2009). Metals being persistent keep accumulating and magnifying with increase in trophic level of food chain. Accumulation of heavy metals beyond permissible limits affects vital organs like, kidneys, bones, liver and blood and causes serious health hazards. Health effects associated with heavy metals like, cadmium, copper, lead and chromium include gastrointestinal effects, renal impairment, neurological disorders, cardiovascular troubles, bone problems, convulsions, paralysis etc. Metals, because of their solubility in water, are toxic and the toxicity can be acute or chronic dependent upon exposure-time (Dorne et al. 2011; Järup 2003). Toxicological studies have found heavy metals to be carcinogenic, teratogenic, mutagenic and neurotoxic (European Union 2002).

Vegetables are important component of human platter because of high nutritional value and antioxidants. Leafy and tuberous vegetables tend to accumulate higher concentration of heavy metals than grains and fruits. Many studies across the globe have reported high content of heavy metals in vegetables cultivated with wastewater (Boamponsem et al. 2012; Flores-Magdaleno et al. 2011; Mathur et al. 2006). Water can percolate and infiltrate from wastewater drain to the adjoining areas and the content of metals in plants growing in the vicinity of wastewater drain can also be significantly elevated which can cause serious repercussions to the society. Present study aims to compare the metal content (copper, cadmium, lead, iron and cobalt) in vegetables irrigated with wastewater and those irrigated with ground water but cultivated across wastewater drain in agricultural sites of Punjab, India. Furthermore comparison of hazard quotient associated with intake of these metals in adults (male and female) and children is also assessed.

## Methods

Experiments were carried out in Research Laboratory of Department of Botanical and Environmental Science of Guru Nanak Dev University, Amritsar, Punjab, India. Samples of 12 common vegetables were collected from agricultural fields across Amritsar. Details of vegetables collected are given in Table 1.

**Table 1:** Vegetable samples collected from experimental sites

Edible part	Vegetable (common name)	Botanical name	Family
Leaf and stem	Coriander	*Coriandrum sativum*	Apiaceae
	Fenugreek	*Trigonella foenum*-*graecum*	Fabaceae
	Mint	*Mentha spicata*	Lamiaceae
	Spinach	*Spinacia oleracea*	Amaranthaceae
Fruits	Brinjal	*Solanum melongena*	Solanaceae
	Bottle Gourd	*Lagenaria siceraria*	Cucurbitaceae
	Lady Finger	*Abelmoschus esculentus*	Malvaceae
	Green Chilli	*Capsicum annuum*	Solanaceae
Tubers	Radish	*Raphanus sativus*	Brassicaceae
	Turnip	*Brassica rapa*	Brassicaceae
Bulbs	Onion	*Allium cepa*	Amaryllidaceae
	Garlic	*Allium sativum*	Amaryllidaceae

### Site description

**Fig. 1:**
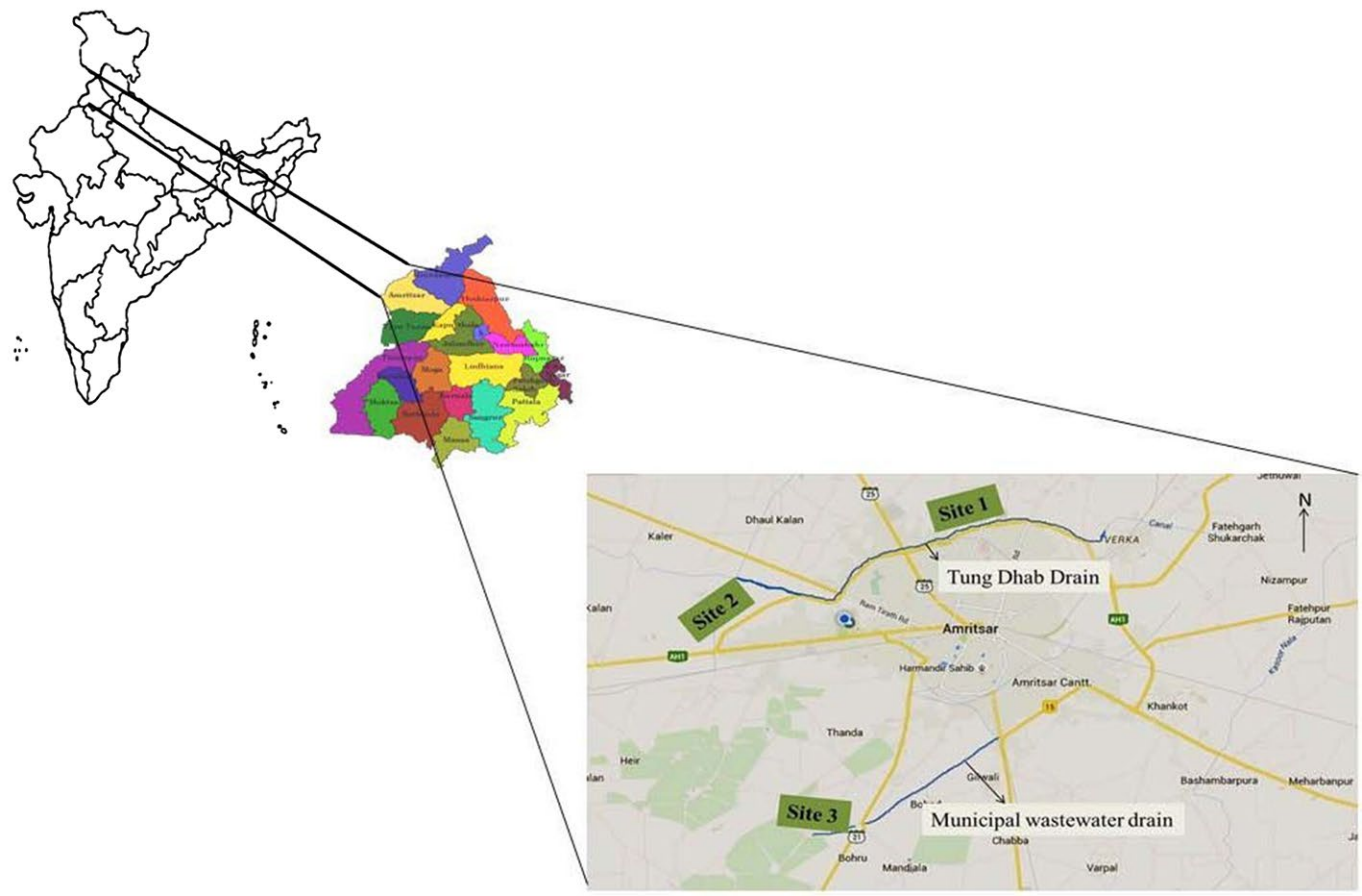
Description of sites

Three sites under vegetable cultivation were selected on the basis of exposure to wastewater. From each site the samples were collected from different fields under vegetable cultivation. The sites are represented in Fig. 1. The drains in consideration are highlighted with blue color.

- *Site 1* Site was located at north-west of Amritsar across the Tung-Dhab drain, and upstream of a Village named Mahal where number of cancer cases is increasing at high rate. This drain carries effluents from two drains (Gumtala drain and Verka drain). The discharge of municipal waste and effluents from various industries (Dairy plant, Paper Mill, Textile industry and Iron foundries) are released into the drain. This site was nearest to the industrial discharges. Agricultural fields at this site are irrigated with ground water.
- *Site 2* Also, located across Tung-Dhab drain and downstream of Village Mahal. The site was 15 km downstream of Site 1. Main source of irrigation at this site was also ground water.
- *Site 3* Site on the southern side of city. Municipal wastewater drain flows across this area and the agricultural fields at this site are irrigated with municipal wastewater.

### Sample collection and preparation

Edible parts of vegetables growing across each site were coded and collected in triplicate. They were brought to laboratory and wrapped in absorbent paper after sufficient washing with distilled water and initial air-drying. Samples were then oven-dried at 70 °C to remove all moisture content. Dried samples were crushed using pestle and mortar.

### Digestion of samples

0.5 g of dried vegetable sample was digested using Tri-Acid mixture (HNO_3_:H_2_SO_4_:HClO_4_ = 5:1:1) till we obtained transparent fumes. Samples were cooled and filtered using Whatman filter paper no. 1. The final volume was made 50 ml using double distilled water. Heavy metal content of samples was analyzed using Atomic Absorption Spectrophotometer (Model: 240FSAA Make: Agilent Technologies).

### Analysis

Standard solution (1000 mg/l) of different metals viz. copper, cobalt, iron, lead and cadmium were procured from Agilent technologies. Standard curve was prepared using various concentrations made from standard solution. Digested samples were then analyzed for the metal content.

### Data analysis

Content of heavy metals in vegetable samples was estimated. Apart from content, following parameters were assessed to estimate risk associated with uptake of metals:

#### Metal pollution index

Assessment of overall load of metals in each vegetable growing at each site was made by computing metal pollution index (MPI) (Usero et al. 1997). It is calculated as the geometric mean of concentration of all metals in edible part of plant.

$$MPI \left( {\text{mg/kg}} \right) = \left( {C_{1 } \times C_{2} \cdots \times C_{n} } \right)^{{\frac{1}{n}}}$$

where C_n_ = Concentration of metal n in sample.

#### Hazard quotient

The screening level risk associated with consumption of contaminated food can be assessed using hazard quotient (US Environmental Protection Agency (US EPA) 1989) Hazard quotient for adults (male and female) and children (below 3 years) associated with the intake of metals along with vegetables from experimental sites was assessed using the following formula:

$$HQ = \frac{{\left( D \right) \times \left( {C_{metal} } \right)}}{{\left( {R_{f } D} \right) \times BO}}$$

where, D = daily intake of food (kg/day), C_metal_ = concentration of metal (mg/kg), R_f_D = reference oral dose of metal (mg/kg of body weight/day) and BO = Body weight (kg). Daily intake of vegetables was taken as 0.100 kg for adults, as this is the minimum vegetable requirement for a balanced diet, it is prescribed that for a balanced diet, one must have a minimum serving of 100 g for 3 times (National Institute of Nutrition 2011). Also, a survey conducted by USDA has suggested that people from countries like India consume 100 g per capita per day of vegetables (Kanungsukkasem et al. 2009). Daily intake of vegetables for children below 3 years was taken as 0.05 kg (National Institute of Nutrition 2011). Average body weight for adult male was taken as 55 kg and average body weight of female as 45 kg and of children (below 3 years) is 12 kg (Indian Council of Medical Research 2010). Reference oral dose (mg/kg/day) for metals is given in Table 2.

**Table 2:** Table showing reference oral dose for metals

S. no.	Metal	Reference oral dose (mg/kg/day)	Reference
1.	Cadmium	0.001	US EPA (2015)
2.	Copper	0.04	US EPA (2015)
3.	Cobalt	0.043	Food and Nutrition Board (2004)
4.	Lead	0.004	US EPA (1997)
5.	Iron	0.7	US EPA (2015)

### Statistical analysis

Statistical significance of variation in content of heavy metals in vegetables from different sites was tested using two way ANOVA followed by post hoc Tukey HSD test for metal concentration in vegetables from different sites. All the statistical calculations were done using SPSS 17.0.

## Results and discussion

### Content of heavy metals in vegetables

**Fig. 2:**
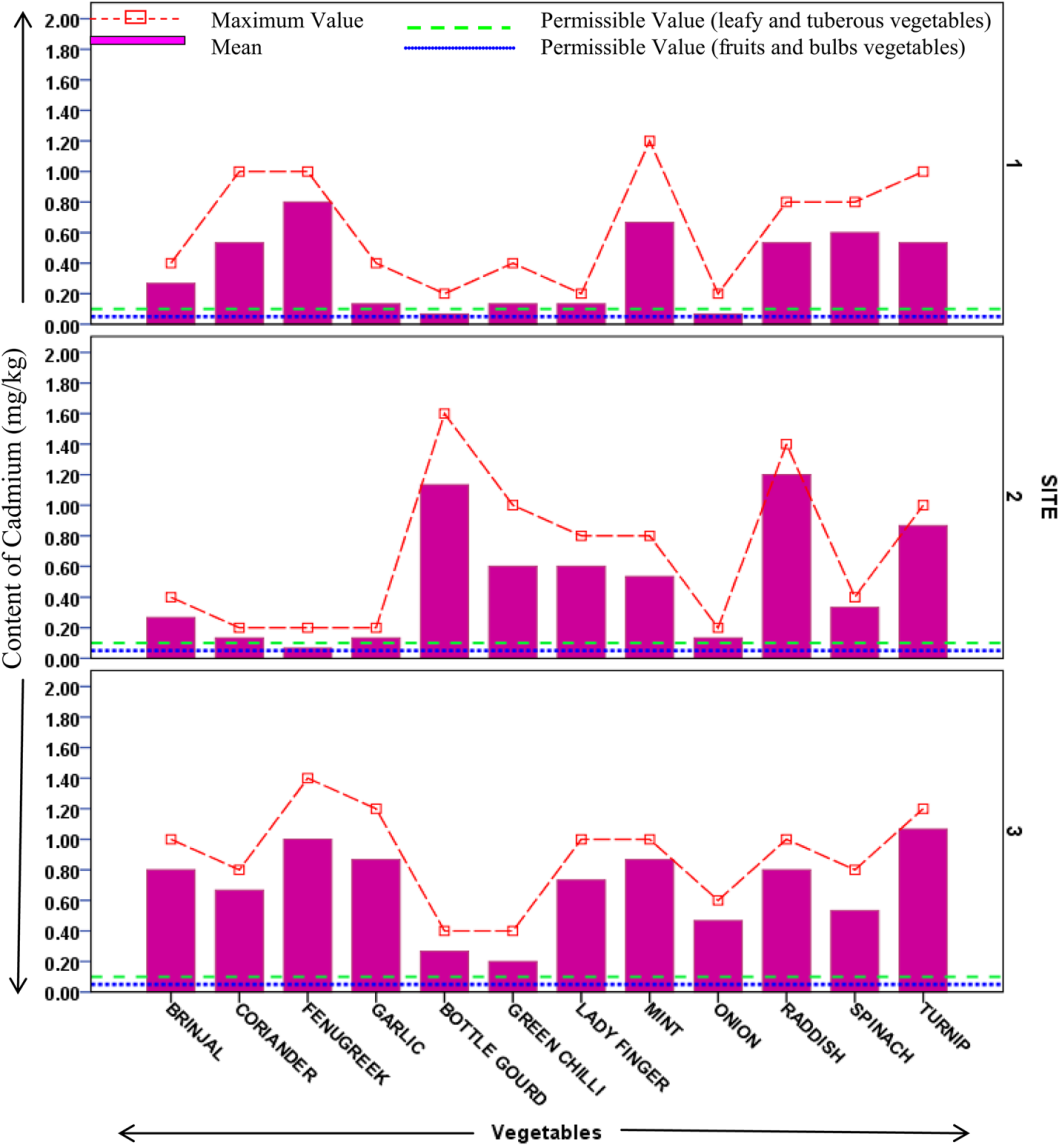
Content of cadmium in vegetables from all the sites

**Fig. 3:**
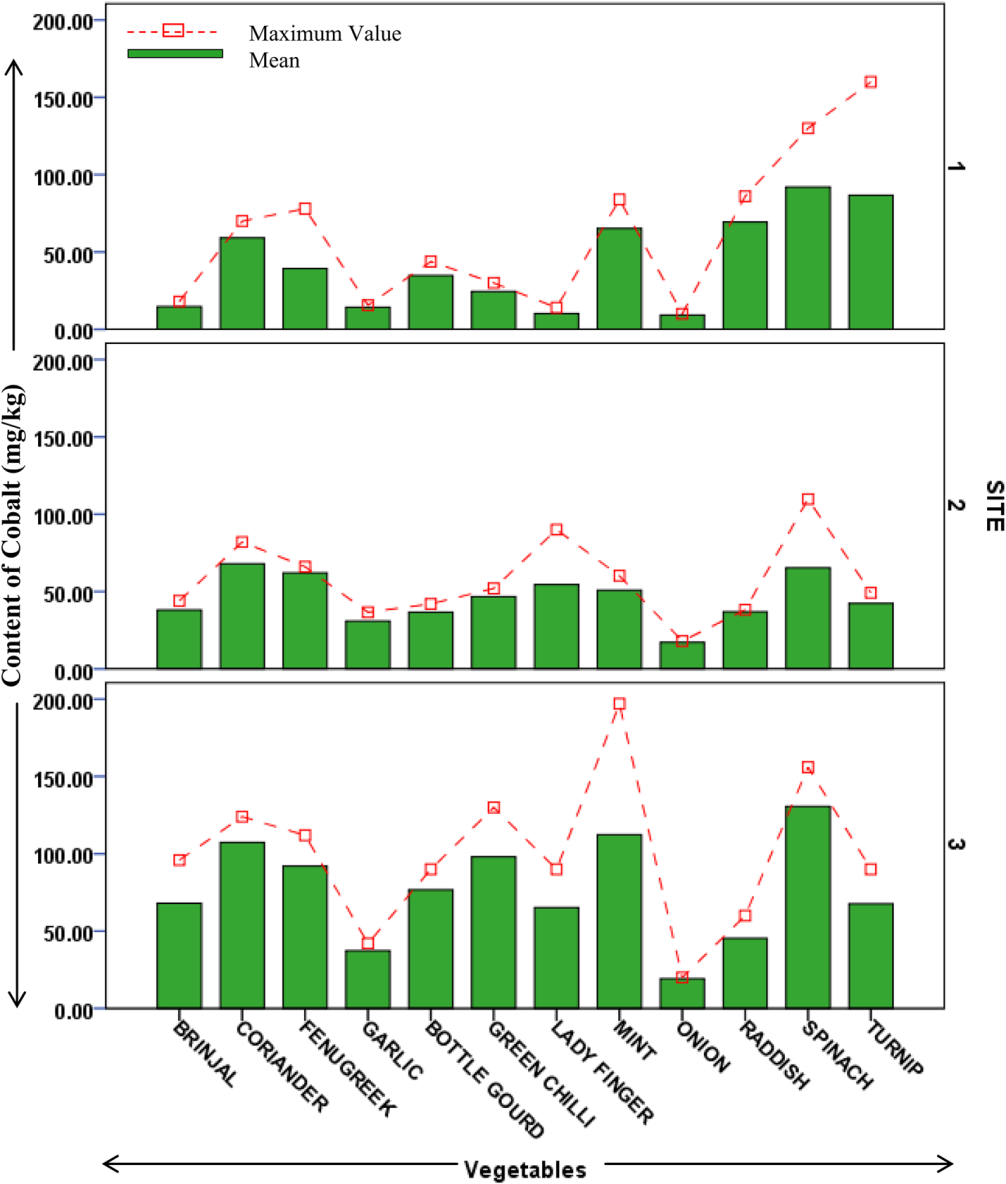
Content of cobalt in vegetables from different sites

**Fig. 4:**
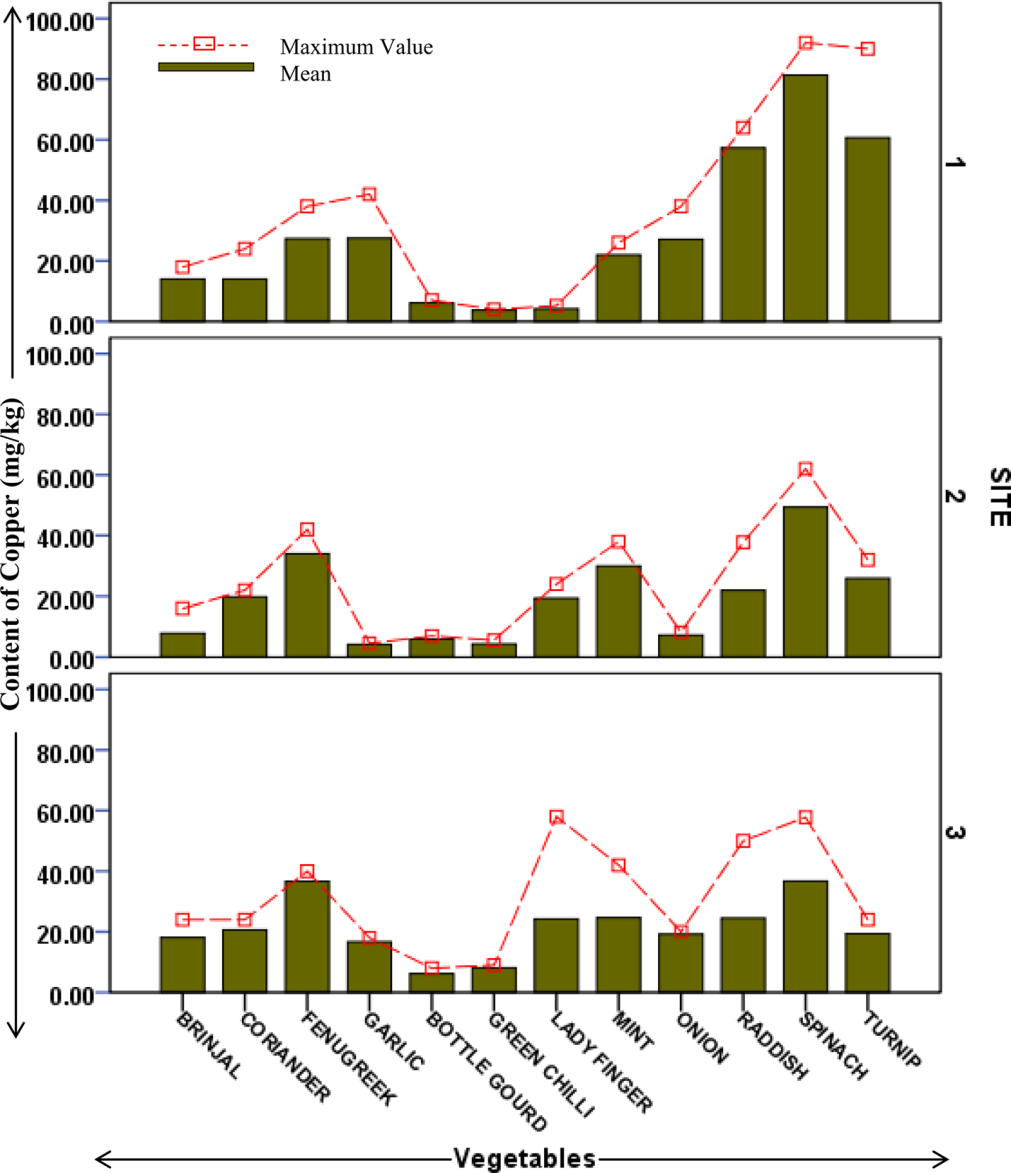
Content of copper in vegetables from different sites

**Fig. 5:**
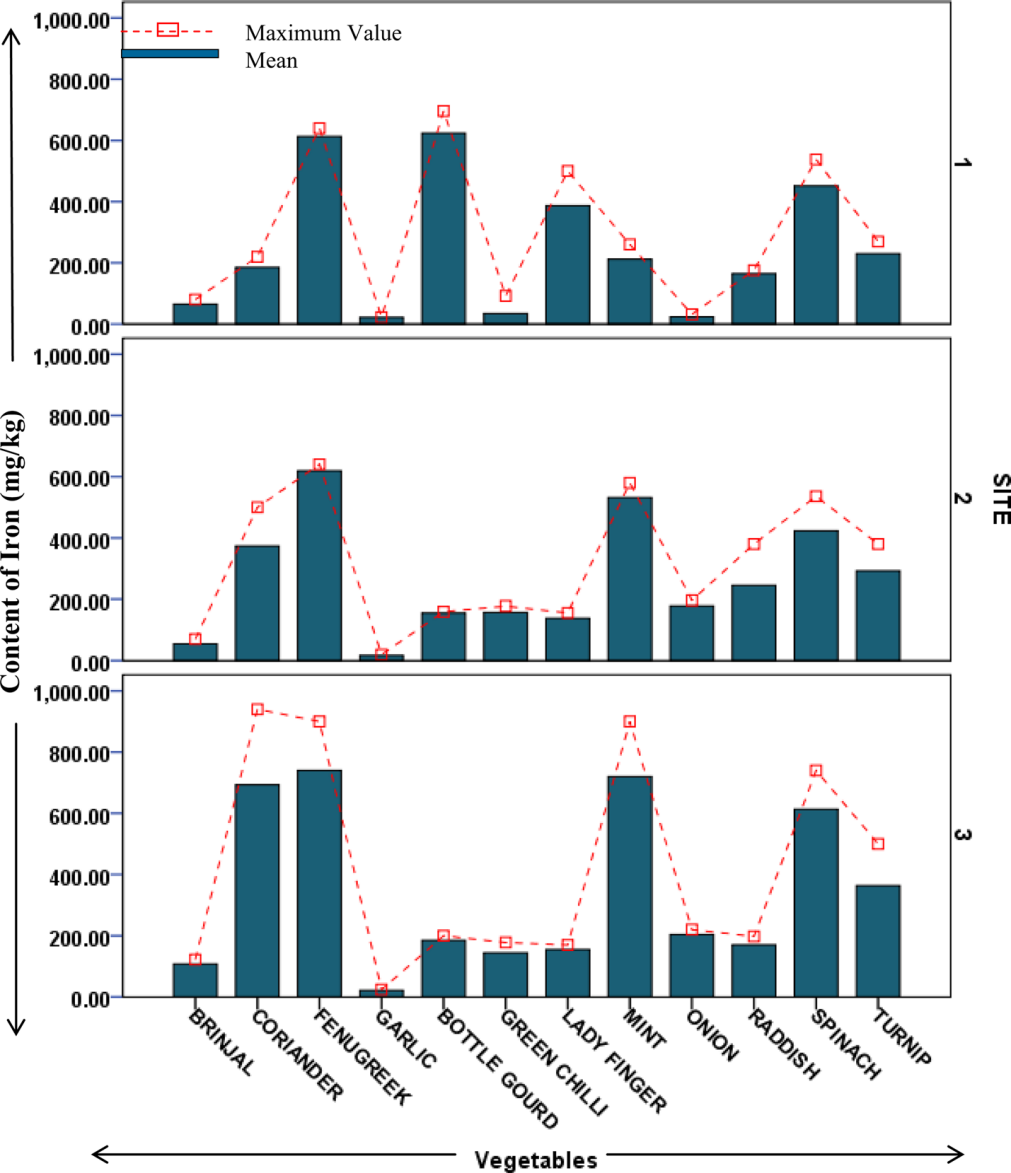
Graph representing content of iron in vegetables from different sites

**Fig. 6:**
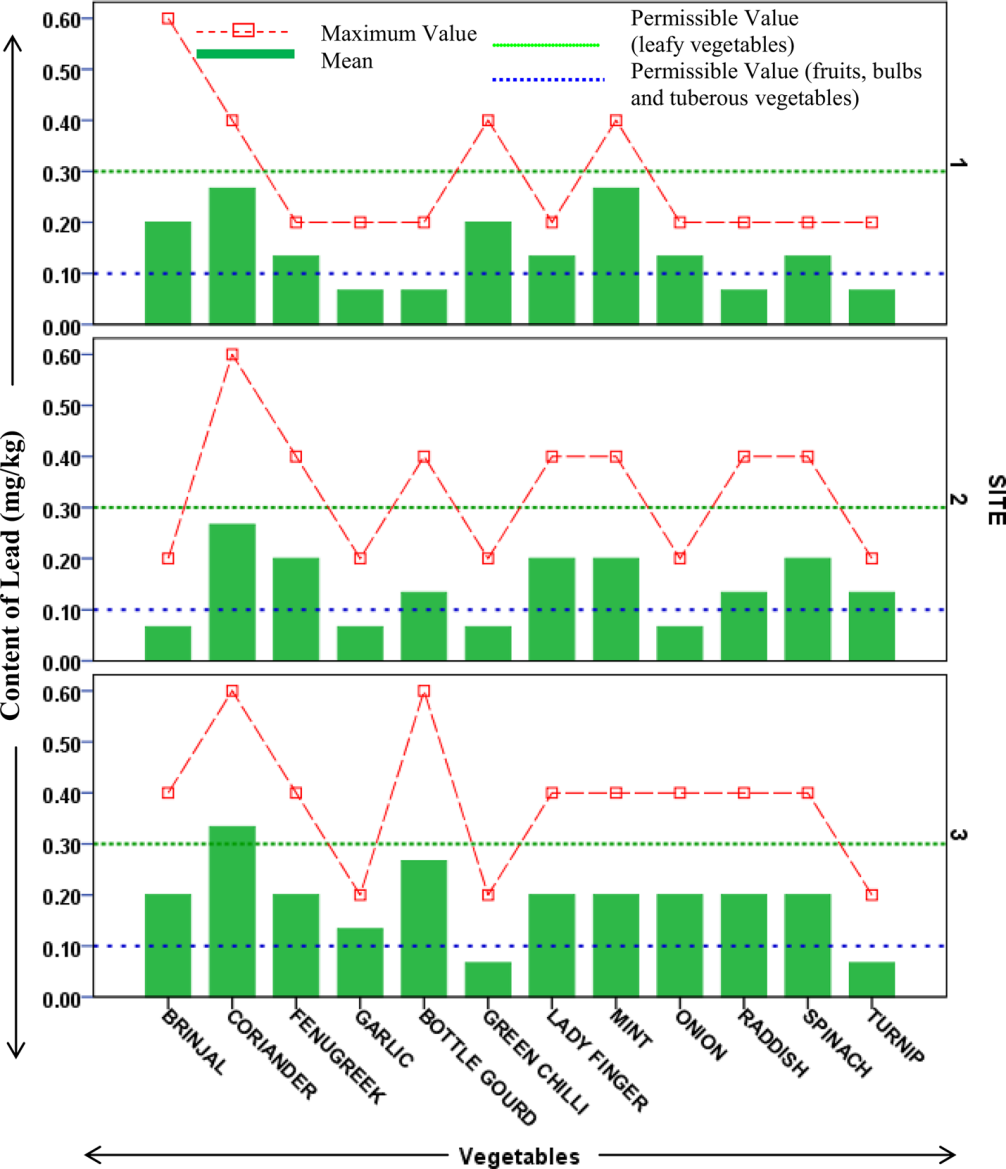
Content of lead in vegetables from different site

Heavy metal contents in different vegetable samples from each site are represented in respective figures. Figure show the mean content of metal in each vegetable and maximum value reported among replicates. Uptake pattern of heavy metals by vegetables was found to be similar in all the sites. In all the three sites the uptake of metals exhibited the following trend: *Iron* > *Cobalt* > *Copper* > *Cadmium* > *Lead*. The concentration of all metals was found to be higher in the leafy and tuberous vegetables as compared to fruit vegetables with an exception of iron in vegetable samples from site 1. This trend was found to be in accordance with previous studies estimating heavy metals in vegetables (Arora et al. 2008; Singh et al. 2010). At Site 1 (irrigated with ground water but located across wastewater drain) uptake of cadmium was found to be maximum in fenugreek (0.8 mg/kg) followed by spinach (0.6 mg/kg). Cadmium is a possible carcinogen and dietary intake of cadmium affects kidneys and liver. Permissible limit for cadmium in bulb and fruits vegetables is 0.05 mg/kg, in leafy and tuberous vegetables is 0.1 mg/kg (FAO/WHO 2014). Mean concentration of cadmium in all samples from all sites was higher than permissible limits. Maximum concentration of cadmium (1.2 mg/kg) was reported in raddish samples from site irrigated with ground water (site 2). Site 2 (away from industrial discharge point) had lesser cadmium concentration in leafy vegetables as compared to those in site 1. At site 2 Turnip contained maximum content of Cd (0.87 mg/kg). Site 3 which is irrigated with wastewater showed maximum uptake of cadmium in turnip (1.06 mg/kg) followed by fenugreek (1 mg/kg) and raddish (0.8 mg/kg), as shown in Fig. 2. Accumulation of cobalt was maximum in spinach samples from both site 1 and site 3 (92 and 130.67 mg/kg, respectively), while being maximum in coriander samples (69 mg/kg) from site 2 (Fig. 3). Among all the sites, the least concentration of cobalt was reported in bulb vegetables. Cobalt though is an essential element, but its excess is known to cause phytotoxic effects in plant and interference in uptake of other essential elements (Nagajyoti et al. 2010). Human intake of higher concentrations of cobalt cause serious toxic effects which are attributed to its affinity to sulfhydryl group or because of antagonistic effects in calcium ion channel (Simonsen et al. 2011). The concentration of copper (80.33 mg/kg) was found highest in spinach samples of site 1 which is irrigated with ground water but growing in vicinity of wastewater drain (Fig. 4). Copper is also an essential element but its higher levels are known to cause toxicity effects and acute exposure (200 mg/kg) can lead to death (FAO/WHO 2011). Arora et al. (2008) has reported the concentration of copper in spinach in range of 15.9–17.4 mg/kg. In present study, it was observed that concentration of copper in ground water irrigated site (in vicinity of wastewater drain) was many folds higher than as reported by Arora et al. (2008). Iron was most accumulated metal among all plant samples. Mean concentration of Iron in samples from site 1 was maximum in bottle gourd (624 mg/kg) followed by fenugreek (612 mg/kg). In samples from site 2 and 3 maximum iron uptake was found in fenugreek (618 and 740 mg/kg). Minimum concentration of iron was reported in garlic samples from all sites (Fig. 5). Permissible concentration of lead in fruit, tuberous and bulb vegetables is 0.1 mg/kg, while that in leafy vegetables is 0.3 mg/kg (FAO/WHO 2014). It was observed mean content of lead in 7 out of 12 vegetable samples from site 3 was higher than permissible limit. Some vegetable samples from site 1 and 2 were found to contain lead higher than permissible limit (Fig. 6). The general trend of uptake of heavy metals revealed higher concentration of metals in leafy and tuberous vegetables as compared to fruits and bulbs.

Results of two way ANOVA revealed that the variation in metal concentration (except lead) is significant with respect to site and type of vegetables (Table 3). Variation in uptake of metals in different sites is attributed to the differences in availability of metals at different sites. Also, the speciation of metals is responsible for the bioavailability of metals at different sites. The variation in uptake of metals with respect to different vegetables is because of difference in morphology, variation in translocation of aqueous solution in plant, difference in physiological parameters of different crops. Summary of post hoc Tukey HSD test are given in Table 4. Results of post hoc Tukey HSD test revealed that there exists no significant difference between concentration of metals viz. cadmium, cobalt and iron in site 1 and site 2 (along the wastewater drain) but concentration of these metals in vegetables from site 3 which is irrigated with wastewater was significantly different (p < 0.01) than concentration of metals in samples from other sites. In case of copper statistically significant difference was observed in samples from site 1 and 2 at p < 0.01 level. Also, concentration of copper in vegetable samples from site 1 and 3 were significantly different (p < 0.05). No statistically significant difference was observed in concentration of copper in samples from site 2 (irrigated with ground water but in vicinity of wastewater drain) and 3 (irrigated with wastewater).

**Table 3:** Two way ANOVA summary table

Metal	Site	Vegetables	Site × vegetables
Cadmium	13.938**	5.023**	4.372**
Copper	7.044*	14.346**	3.193**
Iron	13.106*	51.320**	9.245*
Lead	0.696	0.850	0.269
Cobalt	19.614**	7.240**	1.644

** Level of significance; p < 0.001

* Level of significance; p < 0.01

**Table 4:** Summary of post hoc Tukey HSD test with respect to sites

Site (i)	Site (j)	Cadmium mean difference (i–j)	Cobalt mean difference (i–j)	Copper mean difference (i–j)	Iron mean difference (i–j)
1	2	−0.1278	−2.5056	9.6333*	−14.5556
	3	−0.3167*	−33.3778*	7.5222*	−91.9944*
2	1	0.1278	2.5056	−9.6333*	14.5556
	3	−0.1889*	−30.8722*	−2.1111	−77.4389*
3	1	0.3167*	33.3778*	−7.5222*	91.9944*
	2	0.1889*	30.8722*	2.1111	77.4389*

* Significant at p < 0.01

#### Metal pollution index

**Fig. 7:**
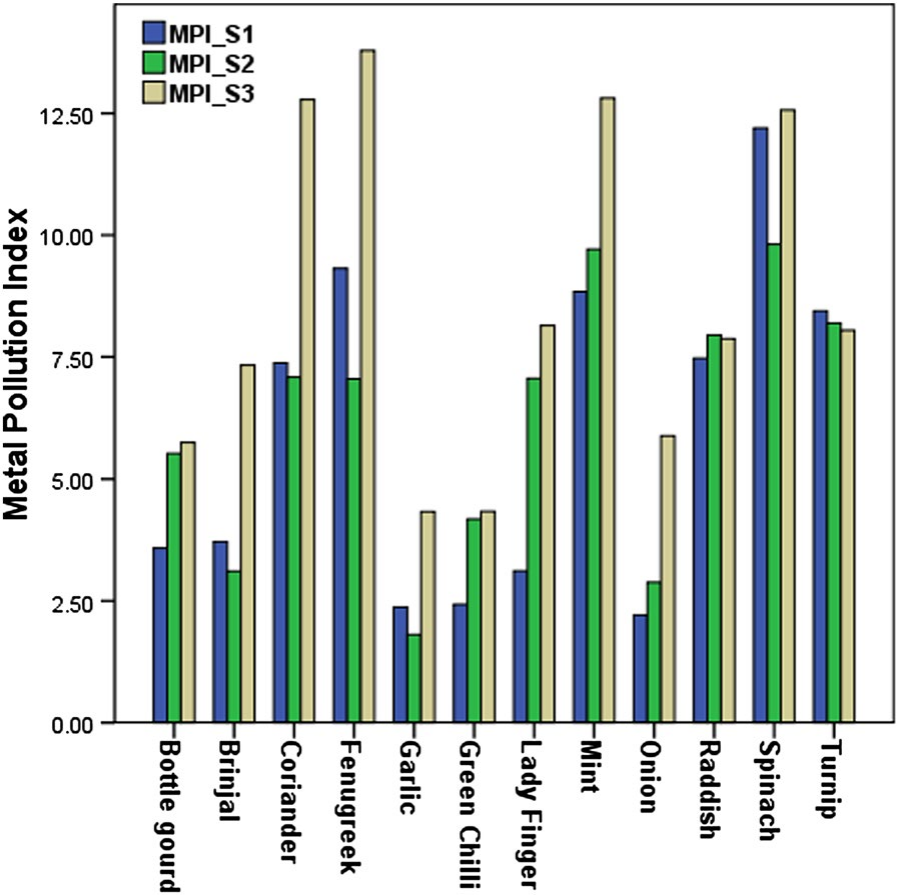
Metal pollution index of vegetables from different sites

Metal pollution in samples is reliably estimated using metal pollution index (MPI). Figure 7 demonstrates metal pollution index of vegetable samples from each site. It was found that metal pollution index of vegetable samples from site-3 which is irrigated with wastewater was higher than that of vegetable samples of other sites (except turnip and raddish). Both the tuberous vegetables i.e. raddish and turnip exhibited very interesting results showing that the metal pollution index of these vegetables from all three sites was similar and in case of turnip site 1 and site 2 samples (both in close proximity of wastewater drain) had higher MPI than site 3. Metal pollution index of leafy vegetables like, fenugreek, coriander, mint and spinach was found to be maximum followed by tuberous vegetables like, raddish and turnip. It is to be observed that metal pollution index in spinach from Site 1 was similar to the metal pollution index of spinach from wastewater irrigated site. The general trend of metal pollution index from site 1 was Spinach > Fenugreek > Mint > Turnip > Raddish > Coriander > Brinjal > Bottle Gourd > Lady Finger > Green Chilli > Garlic > Onion. Also, In case of site 2 among all vegetables spinach exhibited maximum metal pollution index (Spinach > Mint > Turnip > Raddish > Coriander > Lady Finger > Fenugreek > Bottle Gourd > Green Chilli > Brinjal > Onion > Garlic). Trend for vegetable samples from site 3 was: Fenugreek > Coriander > Mint > Spinach > Lady Finger > Turnip > Raddish > Brinjal > Onion > Bottle Gourd > Garlic > Green Chilli.

#### Hazard quotient

**Fig. 8:**
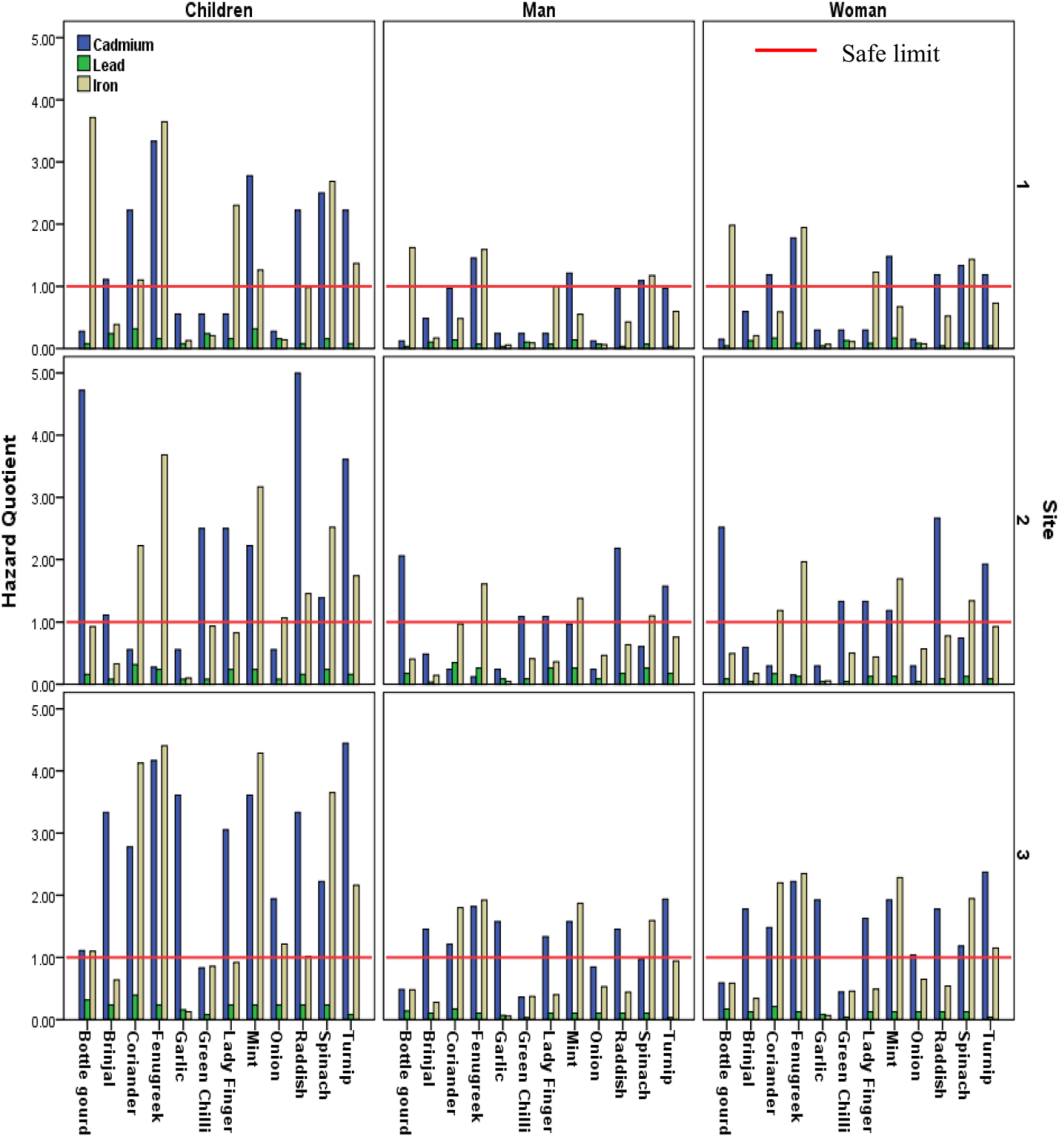
Hazard quotient of cadmium, lead and iron of different vegetables from three sites

Health risk associated with any pollutant is dependent upon the level of exposure and amount of absorption by human body. Thus, hazard quotient is a valid tool to assess the level of risk associated with particular pollutant. If level of Hazard quotient is less than 1, the risk associated with exposure of metal is negligible. However if level of hazard quotient is higher than 1, the metal may pose serious health hazards. The estimation of hazard quotient of metals in different vegetables from various sites demonstrated alarming results. Though the concentration of some metals was significantly higher in vegetable samples from wastewater irrigated sites but the health risk associated with these metals was also found to be very higher. Cadmium identified as potential carcinogen by US EPA (2010), was found to be at hazardous level in most of the vegetable samples. Hazard quotient of cadmium was found to be maximum in raddish samples from site 2 which is close proximity to wastewater drain. It was observed that hazard quotient of cadmium was maximum in tuberous vegetables, followed by leafy vegetables. For children the consumption of 11 out of 12 vegetables from wastewater irrigated site was hazardous while 7 and 8 vegetables also from site 1 and 2 respectively, can be hazardous with respect to cadmium. Concentration of lead was within permissible limits for all samples except bottle gourd and lady finger from site 1 and 3 but the hazard quotient associated with lead was below 1 in all samples for adults and children. Iron was found to be above the safe limits in samples of leafy vegetables. Iron though is vital element for human life can cause severe toxicity symptoms when in excess. Fenugreek samples from all sites exhibited the level of hazard quotient associated with iron was highest among all vegetables. Figure 8 represents hazard quotient for adults and children of cadmium, lead and iron in vegetable samples from all the three sites.

**Fig. 9:**
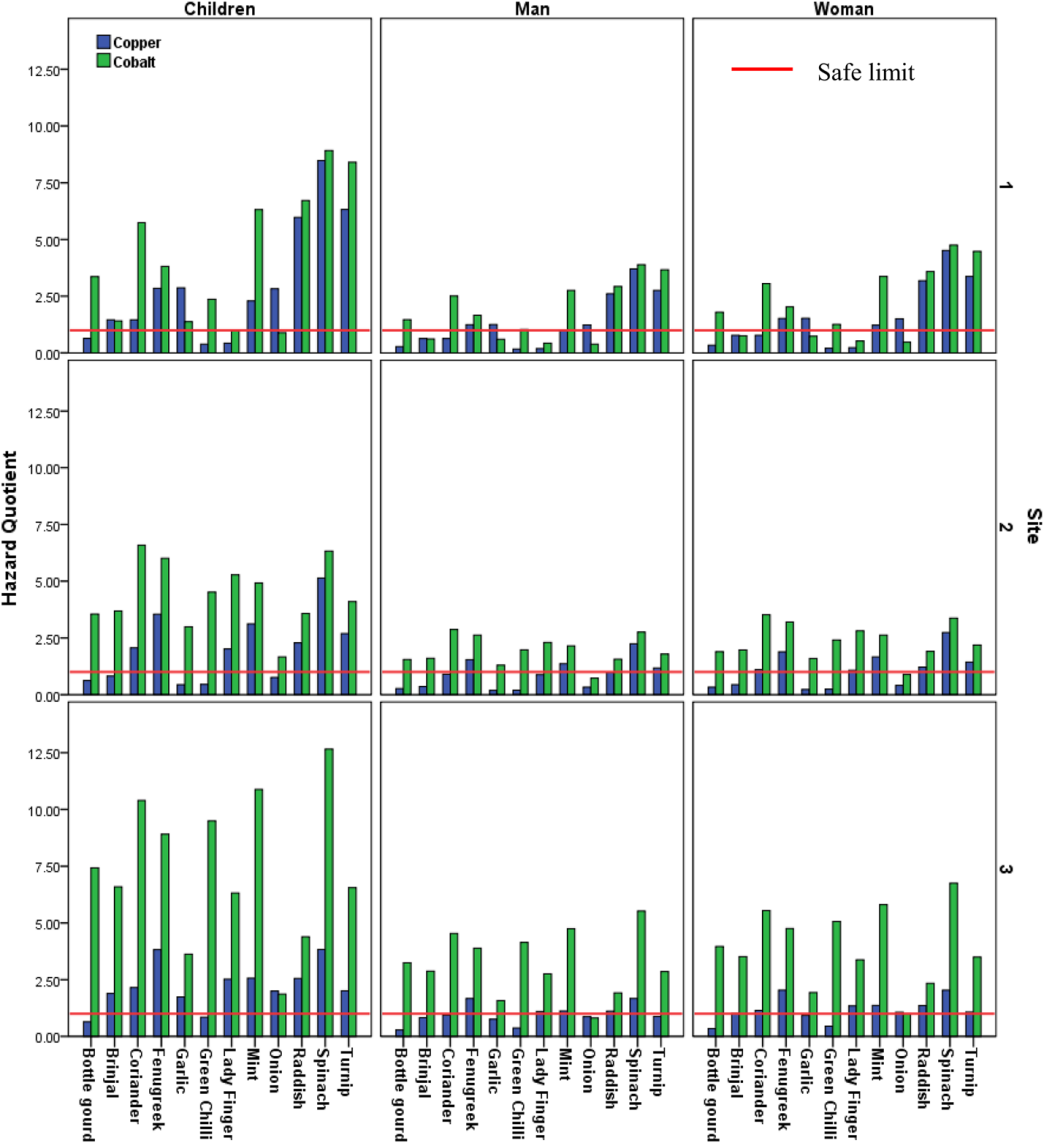
Hazard quotient of cobalt and copper of different vegetables from three sites

Hazard quotient of cobalt was found more than one for children in almost all vegetables except for fruits and bulb vegetables. Cobalt is component of Vitamin B12 and is important for various physiological functions. But toxicity of higher concentration is widely reported (Simonsen et al. 2012). Maximum hazard quotient for cobalt was from the vegetable samples from site 3 (Fig. 9). Spinach samples from site 1 exhibited highest hazard quotient for copper as evident from Fig. 9. Spinach samples from all the sites can be highly hazardous for children. It is to be noted that we calculated hazard quotient using the minimum dietary requirement of vegetables in balanced diet.

Apart from various other toxicity affects, US EPA (2015) identifies cadmium to target kidney, cobalt to target endocrine gland and copper and iron to target gastrointestinal tract. Thus these metals can cause serious health effects at their target organs. The hazard quotient and metal concentration in food crops in sites irrigated with wastewater was in accordance with previous studies (Guerra et al. 2012; Gupta et al. 2010; Masona et al. 2011; Orisakwe et al. 2012; Pedrero et al. 2010; Singh and Agrawal 2010). In the present study, we observed that plants growing in vicinity of wastewater drain also pose a significant threat to human health. Various studies have reported increase in number of cancer cases, DNA damage and high frequency of micronuclei in buccal mucosa in people from a Village Mahal across the wastewater drain (Gandhi and Kumar 2004; Sambyal et al. 2004). This can be attributed to consumption of these vegetables.

**Fig. 10:**
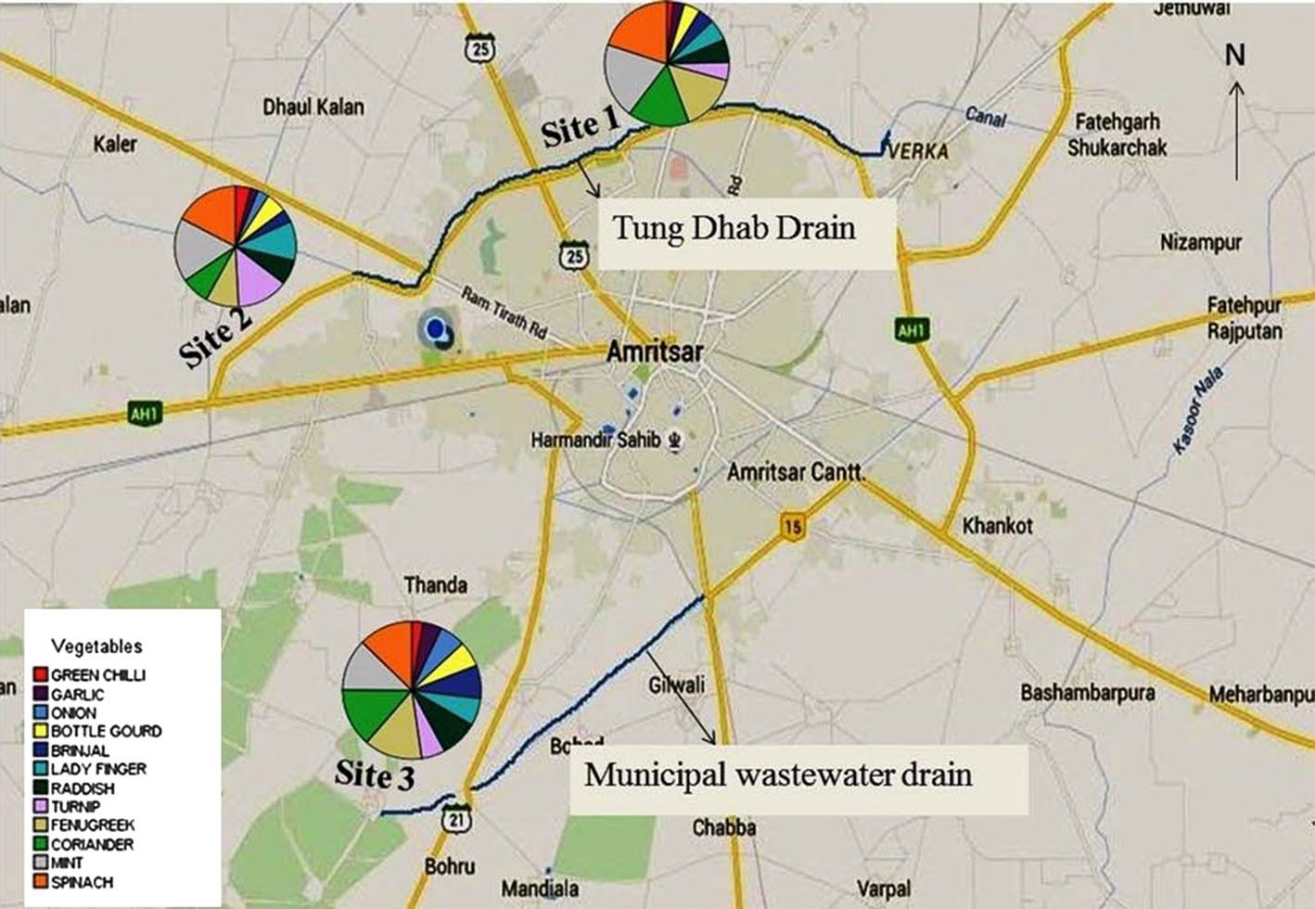
Representation of risk associated with metals present in various vegetables from different sites

Figure 10 summarizes the risks associated with metal content from different vegetables growing on various sites.

## Conclusion

The healthy and balanced diet is considered to be one which is having many servings of vegetables in a day but due to injudicious agricultural practices this can cause a serious threat to human population. The sites irrigated with wastewater due to lack of water resources are the most risky ones but the sites which are in close vicinity with wastewater drains are also of significant consideration due to percolation of water. No significance difference was observed in copper and lead uptake by vegetables growing in sites irrigated with wastewater and those irrigated with ground water but are in close vicinity with wastewater drain. The regular consumption of vegetables grown in sites can cause detrimental effects to the human population. The authors strongly recommend that consumption of tuberous and leafy vegetables from these sites should be restricted and continuous monitoring of vegetables from these sites should be done. Efforts should be made to amend the soil to reduce the uptake of metals in vegetable crops or these sites should be used for cultivation of non-food crops.
